# Bonobos Protect and Console Friends and Kin

**DOI:** 10.1371/journal.pone.0079290

**Published:** 2013-11-05

**Authors:** Elisabetta Palagi, Ivan Norscia

**Affiliations:** 1 Museo di Storia Naturale e del Territorio, Università di Pisa, Calci - Pisa, Italy; 2 Unità di Primatologia Cognitiva, Istituto di Scienze e Tecnologie della Cognizione, Consiglio Nazionale delle Ricerche, Roma, Italy; Max Planck Institute for Evolutionary Anthropology, Germany

## Abstract

Post-conflict third-party affiliation has been reported to have different functional meanings, one of them being consolation. Here, we tested the main hypotheses that have been put forth to explain the presence of this phenomenon at a functional level in the bonobo: *Self-Protection Hypothesis, Victim-Protection Hypothesis, Relationship-Repair* or *Substitute for Reconciliation Hypothesis*, and *Consolation Hypothesis*. By analyzing the data collected over 10 years, we investigated what factors affected the distribution of both spontaneous third party affiliation (initiated by the bystander) and solicited third party affiliation (initiated by the victim). We considered factors related to the individual features (sex, rank, age) of victim and bystander, their relationship quality (kinship, affiliation), and the effect that third party affiliation had on the victim (such as protection against further attacks and anxiety reduction). Both spontaneous and solicited third party affiliation reduced the probability of further aggression by group members on the victim (*Victim-Protection Hypothesis* supported). Yet, only spontaneous affiliation reduced victim anxiety (measured via self-scratching), thus suggesting that the spontaneous gesture – more than the protection itself – works in calming the distressed subject. The victim may perceive the motivational autonomy of the bystander, who does not require an invitation to provide post-conflict affiliative contact. Moreover, spontaneous - but not solicited - third party affiliation was affected by the bond between consoler and victim, being the relationship between consoler and aggressor irrelevant to the phenomenon distribution (*Consolation Hypothesis* supported). Spontaneous affiliation followed the empathic gradient described for humans, being mostly offered to kin, then friends, then acquaintances. Overall, our findings do not only indicate the consolatory function of spontaneous third-party affiliation but they also suggest that consolation in the bonobo may be an empathy-based phenomenon.

## Introduction

“*Given the morphological similarity between ape consolation behavior and expressions of sympathetic concern in young children, which also touch and embrace distressed individuals, we follow the Darwinian principle of parsimony that if two related species show similar behavior under similar circumstances, the psychology behind their behavior is likely similar, too.*”

(De Waal, p. 97, [1])

Empathy, the ability to share emotions, is crucial for many successful social interactions [2]. In humans, empathy comes into play in a vast array of life arenas, from sales and management to romance and parenting, from compassion to political action [3]. Empathy steadily increases according to the emotional closeness of subjects (empathic gradient), being lower between weakly bonded individuals, higher between tightly bonded ones, and highest in kin [4], [5].

In humans, the act of providing comfort via non solicited affiliation offered to a distressed subject is widely accepted as a crucial behavior that can reveal the empathic potential of individuals [6]–[8]. In non-human primates, third party affiliation is behaviourally described as the first affinitive contact occurring between the recipient of an aggression (namely, the victim) and a bystander not involved in the aggression [9]. Third party affiliation can be provided by an individual either following a request of the victim or offered spontaneously [9]–[11]. In non-human apes, the non solicited affiliation provided by a third party is commonly called “consolation” (chimpanzees, *Pan troglodytes* [11]–[18]; gorillas, *Gorilla gorilla* [19], [20]; bonobos, *Pan paniscus* [21], [22]), which implies an actual consolatory function of the affinitive gesture, resulting in victim's anxiety reduction [23]. In chimpanzees, Fraser et al. [17] found that the behavioural indicators of victim's anxiety (self-scratching and self-grooming) were reduced as a result of the post-conflict affiliation provided by a bystander. The same and another study [24] also found that third-party affiliation was provided more frequently by closely bonded partners, who are expected to react more empathetically to each others' distress than weakly bonded ones. In the same species, other studies found moderate [13] or no evidence [16] of the consolatory function of third-party affiliation. Hence, the debate on the relationship between third-party affiliation, consolation and emotional closeness (possibly informing empathy) in apes remains open.

Bonobos can assist in drawing a clearer picture of the possible function of third-party affiliation in apes. In fact, bonobos show: high levels of reciprocal help, with two or more individuals working together when facing problem solving tasks [25]; xenophilia [26], expressed by high levels of positive interactions with new group members; tolerance [27] related to more socially symmetrical relationships, with higher level of undecided conflicts and absence of formal submission displays [28]–[30]; and male-female co-dominance [31], with a hierarchy not tightly structured. All these features indicate that the bonobo society is characterized by high levels of cooperation and egalitarism, favoring social intervention by third parties as predicted by the *Social Constraints Hypothesis* [32]. Additionally, bonobos show a developmental delay with respect to social play [28], [33], [34] and high levels of non-conceptive socio-sexual interactions [35], [36], two behaviors extensively used to guarantee the homeostasis of the social tension in the group [37], [38]. Finally, compared to chimpanzees, bonobos seem to possess a greater amount of grey matter in the brain regions involved in perceiving others' distress, an emotional state underpinning empathic abilities [39]. Clay and de Waal [22] found that bonobos respond to distress of other group members and provided some - but not definite - support of the possible anxiety reducing effect of third-party affiliation, whose occurrence was biased toward mother-reared juveniles.

The bonobo is a suitable species to test different predictions applying to the hypothesis that consolation can have an actual consolatory function possibly linked to empathy.

In this study we explore the main possible functions envisaged to explain third party affiliation, corresponding to four hypotheses, listed below along with the predictions that can be derived from them.

The *Self-Protection Hypothesis* predicts that third party affiliation is aimed at protecting the bystander from redirected aggression (chimpanzees [40]; macaques [41]; mandrills [42]). If this hypothesis is in place we should find evidence of the redirection phenomenon – with the victim of an aggression attacking another group member - in the social group (Prediction 1a). If Prediction 1a is supported, the bystanders affiliating more with the victim of an aggression should have a higher risk of redirection (Prediction 1b). Finally, bystander affiliation should reduce the likelihood of redirection (Prediction 1c).

The *Victim-Protection Hypothesis* - a new hypothesis never tested before - predicts that third-party affiliation can lower the probability for the victim to receive further aggression by other group members. If this hypothesis is valid we should find evidence of further aggression towards the victim (Prediction 2a) and such aggression (which can be performed by all group members including the former aggressor) should be reduced as a consequence of third party affiliation (Prediction 2b).

The *Relationship-Repair Hypothesis* (chimpanzees [13]) or *Substitute for Reconciliation Hypothesis* (mandrills [42]; chimpanzees [11]) predicts that third party affiliation functions to restore the relationship between former opponents. According to this hypothesis – supported by studies on vervet monkeys [43] and baboons [44] showing kin-mediated reconciliation - third party affiliation toward the victim should occur more frequently by individuals that are closely bonded or kin-related to the aggressor [13], [42] and whose relationship with the victim is irrelevant [13]. In fact, a closely bonded partner of the aggressor is supposed to be more able to restore the relationship with the victim, on the aggressor's behalf. As a result, affiliation from an aggressor's closely bonded partner (or kin) should reduce the likelihood of renewed attack by the same aggressor [42]. Consequently, aggression-induced anxiety is reduced in the affiliation recipient (in this case the victim [13]). Hence, if the *Relationship-Repair Hypothesis* applies to third-party affiliation in bonobos, we should find the phenomenon to be more frequent in absence of reconciliation (Prediction 3a). Moreover, the bystanders affiliating with the victim should be tightly bonded or kin related with the former aggressor (Prediction 3b). If Prediction 3b is supported, renewed aggression by the same aggressor over the victim - if frequent enough - should be reduced after the affiliation (Prediction 3c), and victim's aggression-related anxiety eventually reduced (Prediction 3d).

Finally, according to the *Consolation Hypothesis*, third party affiliation actually has a consolatory function, thus deserving the label “consolation”. As the Relationship-Repair Hypothesis, the Consolation Hypothesis foresees a reduction of victim's anxiety but the source of such benefit is completely different. In this case the affinitive contact should be primarily received from a victim's closely bonded/related partner whereas the strength of the bystander's bond to the aggressor should be irrelevant [13]. Victim's anxiety would ultimately decrease because closely bonded partners are supposed to be more effective in relieving distress, due to their empathetic connection with the victim [11], [13], [17], [32]. Social closeness, indeed, is one of the main components of the emotional structure of empathy, in accordance with the perception-action model (PAM) proposed by Preston and de Waal [4]. Hence, if the *Consolation Hypothesis* is valid, the bystanders providing affiliation should be tightly bonded or kin related with the victim (Prediction 4a) and victim's aggression-related anxiety should be reduced after the affiliation (Prediction 4b).

## Results

Via the Post Conflict-Matched Control method (PC-MC [45]) we evaluated the presence of reconciliation, spontaneous and solicited third party affiliation (see Methods for details). The presence of reconciliation and third-party affiliation can be demonstrated by comparing the distribution of attracted, dispersed and neutral pairs, calculated for each subject and over all PC-MC pairs. Pair types are defined as follows. In attracted pairs, affinitive contacts (between the opponents, reconciliation; between victims and bystanders, third party affiliation) occurred earlier in the PC than in the MC (or they did not occur at all in the MC), whereas in dispersed pairs the affinitive contacts occurred earlier in the MC than in the PC (or they did not occur at all in the PC). In neutral pairs, affinitive contacts occurred during the same minute in the PC and the MC, or no contact occurred in either the PC or the MC. Then we measured the Corrected Contact Tendency (CCT, reconciliation) and Triadic Contact Tendency (TCT, third party affiliation), per individual victim, defined as the number of attracted minus the number of dispersed pairs divided by the total number of PC–MC pairs [46].

Reconciliation was present in the group thus confirming previous findings [21] (attracted > dispersed pairs; exact Wilcoxon's T = 9.50; N = 15; p = 0.002; TCT = 22.13% ±6.04 SE).

Moreover, we confirmed the presence of both spontaneous (attracted > dispersed pairs; exact Wilcoxon's T = 0.00; N = 15; p = 0.0001; TCT = 33.71% ±7.19 SE) and solicited third party affiliation (attracted > dispersed pairs; exact Wilcoxon's T = 3.00; N = 15; p = 0.003; TCT = 24.69% ±6.90 SE) in the Apenheul bonobo colony. For the individuals that had received both high and low intensity attacks (N = 12), we could evaluate the effect of aggression intensity (by comparing the high and low intensity conditions) on the frequency of third-party affiliation. Aggression intensity did not significantly affect the occurrence of both solicited (exact Wilcoxon's T = 19.00; N = 12; p = 0.734) and spontaneous (exact Wilcoxon's T = 25.00; N = 12; p = 0.520) third-party affiliation.

### Frequency of redirection

In the study groups redirection was virtually absent. In fact, only two subjects, LO and MW (see Table 1 for group composition) showed the phenomenon once. The redirection frequencies (redirection bouts weighed over the number of times the subject was victim of an aggression) were 0.04 for LO and 0.02 for MW.

**Table 1:** Composition of the study bonobo group hosted at the Apenheul Primate Park (Apeldoorn, The Netherlands) in the different periods of observation.

Subject	Year of birth	Sex	Period A (2000)	Period B (2002)	Period C (2002–2003)	Period D (2009)	Kin relation
Ha	1989	M	X				
Mb	1981	M	X	X	X	X	
Mw	1985	M	X	X	X		
Mo	1985	F	X	X			
J	1985	F	X	X	X	X	
Li	1997	F	X	X	X	X	Z's daughter
Lo	1992	F	X	X	X		
Z	1990	F	X	X	X	X	
K	1999	F	X	X	X	X	Mo's daughter
T	1998	M	X	X			J's son
R	1989	F	X				
Ja	2003	F			X		Lo's daughter
Ln	2003	F				X	J's daughter
H	1978	F				X	
Ho	2006	M				X	H's son
N	2006	F				X	Li's daughter
Za	1998	M				X	H's son
Ya	2009	F				X	K's daughter
Ma	2009	M				X	Z's son

A (July-October 2000), B (April-July 2002), C (September 2002-June 2003), and D (August-October 2009). Over time the group consisted of 8-12 individuals, including two to three adult males, four to six adult/subadult females, and three to five immature subjects (<6 y.o.).

### Third party affiliation and reconciliation

Spontaneous third party affiliation was significantly more frequent in absence (mean ±SE: 0.567 ±0.102) than in presence (mean ±SE: 0.091 ±0.043) of reconciliation (exact Wilcoxon's T = 0.00; N = 0; p = 0.004). Similarly, solicited third party affiliation was significantly more frequent in absence (mean ±SE: 0.288±0.095) than in presence (mean ±SE: 0.071±0.029) of reconciliation (exact Wilcoxon's T = 0.00; N = 9; p = 0.009). All the analyses on third-party affiliation (reported below) were carried out excluding the records in which reconciliation was present.

### Third party affiliation, aggressor and bystander features, and relationship quality

Via LMM, we evaluated which variables could explain the variation in the frequency of spontaneous third party affiliation (Table 2 and 3).

**Table 2:** Description of the variables used in the LMM analyses.

NAME	TYPE
**DEPENDENT VARIABLE**	
Frequency of spontaneous third party affiliation	Scale
Frequency of solicited third party affiliation	Scale
**FIXED EXPLANATORY VARIABLES**	
**Individual characteristics**	
Rank	Categorical (1 = high; 2 = medium; 3 = low)
Sex	Dichotomous (1 = male; 0 = female)
Age	Dichotomous (1 = adult; 0 = immature)
**Relationship characteristics**	
Relationship quality	Scale
**RANDOM VARIABLES**	
Aggressor's, Victim's & Bystander's Identity	Nominal
Period	Nominal

**Table 3:** Best LMM explaining the frequency of spontaneous and solicited third party affiliation as a function of the relationship quality between aggressor and bystander features and relationship quality.

Aggressor/Bystander				
**Spontaneous third party affiliation (AICc = −7.821)**				
**Variables**	**Numerator df**	**Denominator df**	**F**	**p**
Intercept	1.00	14.050	31.826	<0.001
Rank of the aggressor	2.00	45.942	13.975	<0.001
**Random variables**	**Variance**			
Aggressor*Bystander Identity	0.001			
Period	0.069			
**Solicited third party affiliation (AICc = 19.659)**				
**Variables**	**Numerator df**	**Denominator df**	**F**	**p**
Intercept	1	28.681	24.539	<0.001
Aggressor's rank	1	42.603	3.905	0.055
**Random variables**	**Variance**			
Aggressor*Bystander Identity	0.032			
Period	0.000			

Aggressor's and bystander's sex, rank (and aggressor and bystander combination for each variable), kinship, and affiliation levels were entered as fixed factors. This analysis involved only those dyads (*n* = 51) where spontaneous third party affiliation had occurred (occasion opportunities≥3). Only aggressor's rank (Table 3) remained in the best model (best model AICc = −7.821; nearest best model AICc = −1.978; worst model AICc = 15.687). Spontaneous third party affiliation was lower when the aggressor was a high ranking subject (estimated marginal mean ±SE: low rank, 0.300 ±0.073; high rank, 0.057 ±0.070).

Finally, we verified which variables could explain the variation in the frequency of solicited affiliative contacts (Table 2 and 3). Aggressor's and third party's sex, rank (and aggressor and third party combination for each variable), kinship, and affiliation levels were entered as fixed factors. This analysis involved only those dyads (*n* = 48) where solicited contacts had occurred (occasion opportunities≥3). The best model (best model AICc = 19.659; nearest best model AICc =  20.058 only including the intercept; worst model AICc = 23.027) included the aggressor's rank only (Table 3). In particular, solicited contacts were lower when the aggressor had a higher ranking position (estimated marginal mean ±SE: high rank, 0.139 ±0.053; low rank, 0.290 ±0.063).

### Third party affiliation, victim and bystander features, and relationship quality

Via Linear Mixed Model (LMM), we evaluated which variables could explain the variation in the frequency of spontaneous third party affiliation (Table 2 and 4).

**Table 4:** Best LMM explaining the frequency of spontaneous and solicited third party affiliation as a function of the relationship quality between victim and bystander.

Victim/Bystander				
**Spontaneous third party affiliation (AICc = −10.547)**				
**Variables**	**Numerator df**	**Denominator df**	**F**	**p**
Intercept	1.00	13.00	307.313	<0.001
Relationship quality	43.00	13.00	7.211	<0.001
**Random variables**	**Variance**			
Victim*Bystander Identity	0.002			
Period	0.001			
**Solicited third party affiliation (AICc = −62.240)**				
**Variables**	**Numerator df**	**Denominator df**	**F**	**p**
Intercept	1	4.067	23.603	0.008
**Random variables**	**Variance**			
Victim*Bystander Identity	0.003			
Period	0.002			

**Figure 1:**
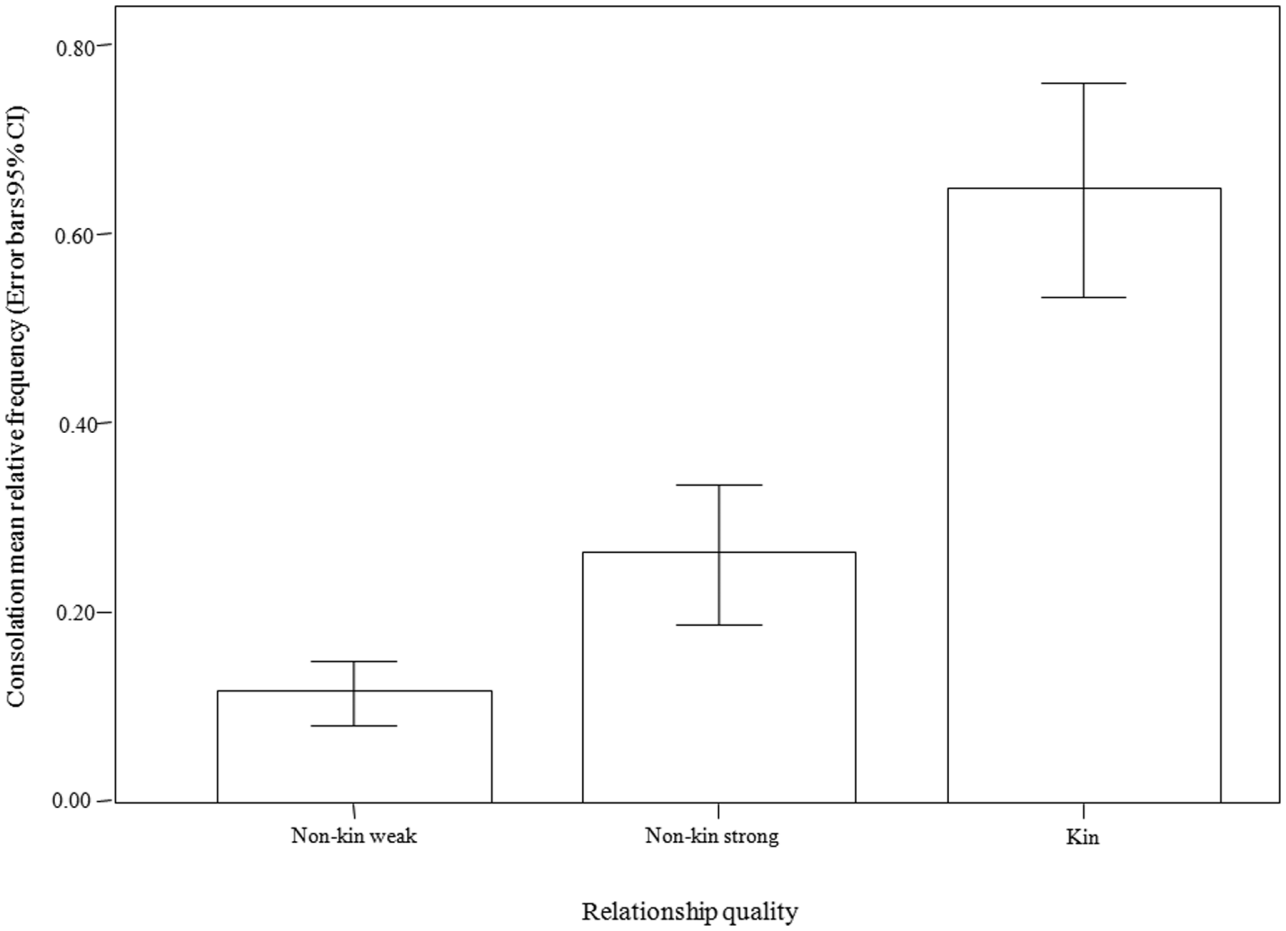
Consolation frequency and relationship quality. Bar graphs of consolation frequency (number of spontaneous third party affiliation events normalized on the number of opportunities, which equaled the number of PCs in which one individual was the recipient, excluding those in which the third-party was involved in the conflict) for bonobo dyads falling into three relationship quality categories: Non-kin weak; Non-kin strong; Kin (with r≥0.25).

Victim's and bystander's sex, age, and rank (and victim and bystander combination for each variable), and relationship quality were entered as fixed factors. This analysis involved only those dyads (*n* = 57) where spontaneous third party affiliation had occurred (occasion opportunities≥3). The best model (best model AICc = −10.547; nearest best model AICc = −6.549; worst model AICc = −0.880) included only the relationship quality (Table 4). The rate of spontaneous third party affiliation was greatest between kin (estimated marginal mean ±SE: 0.633±0.052), then non-kin members with strong relationships (estimated marginal mean ±SE: 0.248±0.035), and lastly non-kin members with weak relationships (estimated marginal mean ±SE: 0.121±0.026) (Figure 1). This gradient is confirmed by randomization tests detecting an extremely significant difference between weakly and strongly bonded, non-kin dyads (randomization test for two independent samples t = −6.414, p = 0.0004) and between kin and either weakly bonded, non-kin members (randomization test for two independent samples t = −11.537, p = 0.00001) or strongly bonded, non-kin members (randomization test for two independent samples t = −4.127, p = 0.0002).

We also verified which variables could explain the variation in the frequency of solicited contacts (Table 2 and 4). Individuals never rejected to provide an affiliative contact when they were asked for it by the victim of an aggression. Victim's and third party's sex, age, and rank (and victim and third party combination for each variable), and relationship quality were entered as fixed factors. This analysis involved only those dyads (*n* = 45) where solicited contacts had occurred (occasion opportunities≥3). None of the factors had an effect on the levels of solicited contacts. Only the intercept (Table 4) remained in the best model (best model AICc = −62.240; nearest best model AICc = −57.569; worst model AICc = −3.223).

### Spontaneous and solicited third party affiliation, and scratching levels

**Figure 2:**
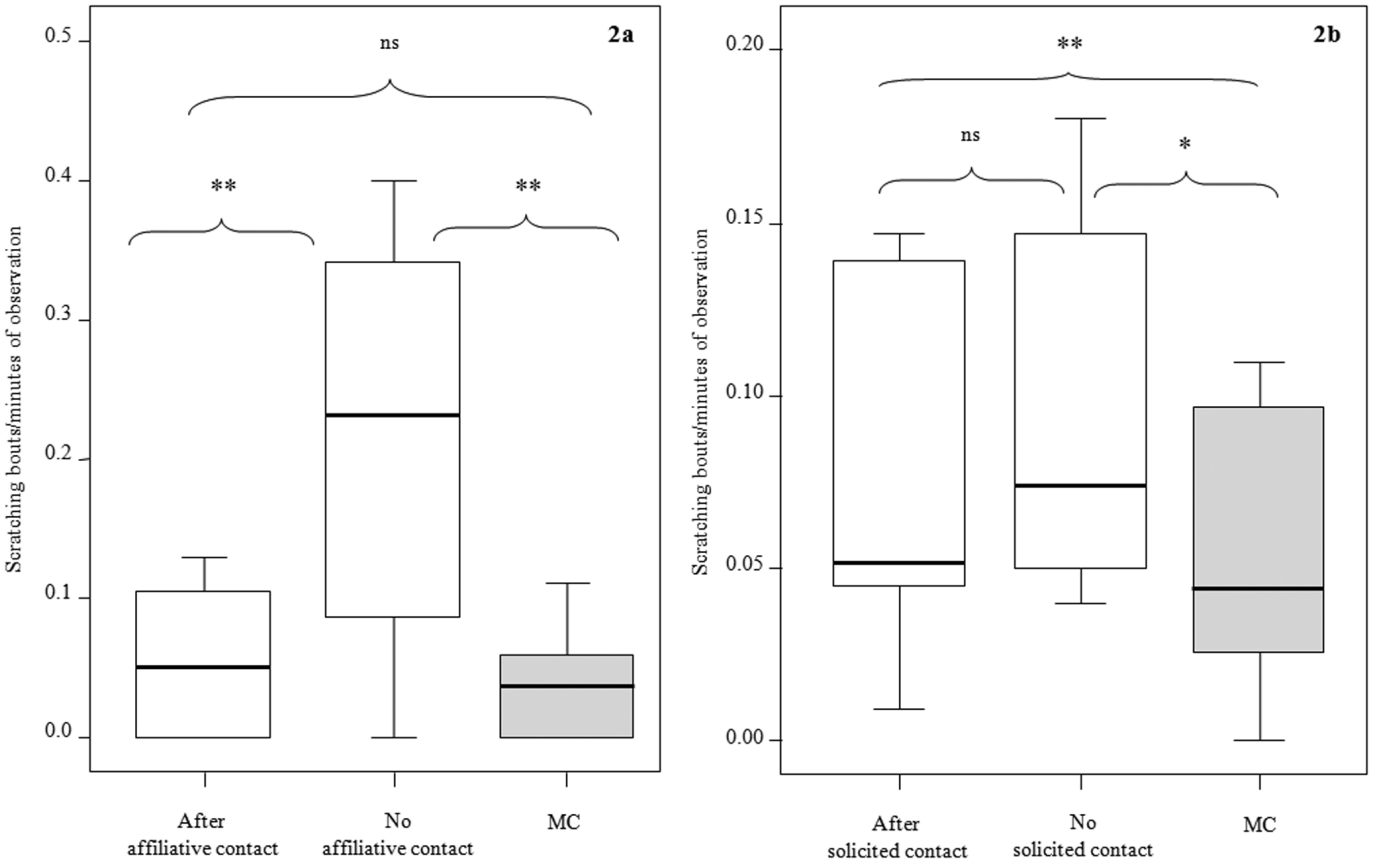
Anxiety related scratching and third party affiliation. Box plots of scratching frequency: 2a) after spontaneous affiliative contact, in absence of affiliative contact and under control conditions (MC); 2b) after solicited affiliative contact, in absence of affiliative contact and under control conditions (MC). Solid horizontal lines indicate medians; box length corresponds to the interquartile range; thin horizontal lines indicate the observed value range. Double and single asterisk, and “ns” indicate highly significant (p<0.01, **), significant (p<0.05, *) and non-significant results respectively. Results were obtained via Dunnett's post-hoc test.

Rough scratching rates were significantly different in the three conditions: without contact (PC-no cont), following spontaneous affiliative contact (PC-spont; in absence of reconciliation), and baseline condition (MC) (Exact Friedman: Chi-square = 12.235, df = 2, N = 10, p<0.001). In particular, compared to MC, scratching rates increased significantly after aggression not followed by affinitive contacts (MC vs PC-no cont; Dunnett's test: q = 3.82, p<0.01). Spontaneous affiliative contacts significantly reduced scratching levels after aggression (PC-spont vs PC-no cont; Dunnett's test: q = 3.33, p<0.01), restoring scratching baseline levels (PC-spont vs MC; Dunnett's test: q = 1.33, ns) (Figure 2a).

Scratching rates were significantly different in the three conditions: without contact (PC-no cont), following the solicited contact (PC-sol; in absence of reconciliation), and baseline condition (MC) (Exact Friedman: Chi-square = 6.228, df = 2, N = 9, p = 0.045). In particular, compared to MC, scratching rates increased significantly after aggression, either followed (MC vs PC-sol; Dunnett's test: q = 2.85, p<0.01) or not followed by affinitive contacts (MC vs PC-no cont; Dunnett's test: q = 2.60, p<0.05). Scratching levels did not significantly differ between PC-no cont and PC-sol (Dunnett's test: q = 0.33, ns) (Figure 2b).

### Spontaneous and solicited third party affiliation, and further aggression on the victim by group members

**Figure 3:**
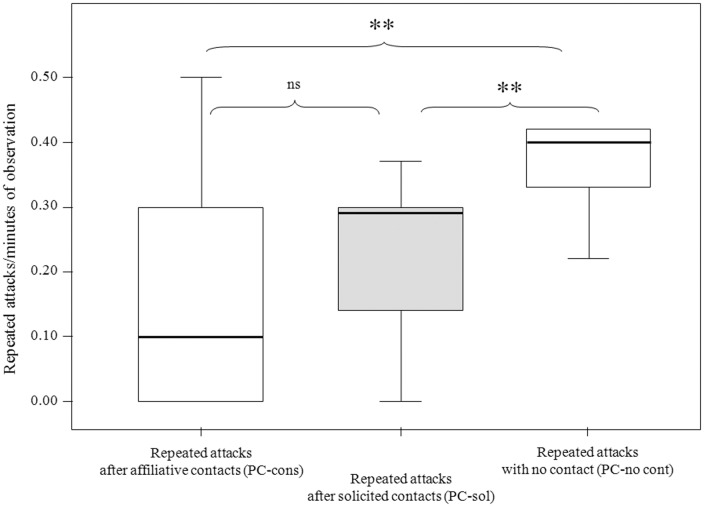
Repeated aggression in presence and absence of third party affiliation. Box plots of frequency of the repeated aggression towards victims after spontaneous affiliative contact (PC-cont), after solicited affiliative contact (PC-sol), and in absence of affiliative contacts (PC-no cont). Solid horizontal lines indicate medians; box length corresponds to the interquartile range; thin horizontal lines indicate the observed value range. Double asterisk and “ns” indicate highly significant (p<0.01, **) and non-significant results respectively. Results were obtained via Dunnett's post-hoc test.

The rates of repeated attacks were significantly different in the three conditions: no contact (PC-no cont), after spontaneous affiliative contacts (PC-spont; in absence of reconciliation), and after solicited contacts (PC-sol; in absence of reconciliation) (Exact Friedman: Chi-square = 13.412, df = 2, N = 9, p<0.0001). In particular, compared to PC-no cont, aggression rates were significantly lower in both PC-spont (Dunnett's test: q = 3.54, p<0.01) and in PC-sol (Dunnett's test: q = 3.18, p<0.01). The aggression rates did not significantly differ between PC-spont and PC-sol (Dunnett's test: q = 2.01, ns) (Figure 3).

## Discussion

Via testing the main alternative hypotheses on post-conflict triadic affiliation, we showed that spontaneous - in contrast to solicited - third party affiliation has a consolatory function in bonobos.

The *Self-Protection Hypothesis* predicts redirected aggression towards the bystander to be reduced after the bystander has affiliated with the victim. Yet, redirection was virtually absent in the study group (Prediction 1a not supported). Consequently, we had to reject the *Self-Protection Hypothesis* as one of the potential explanations of both solicited and spontaneous triadic affiliation in bonobos. This result contrasts with the function of third party affiliation found in mandrills (*Mandrillus sphinx*) [42]. The authors found that victims received most affiliation from those bystanders that were frequently the target of redirection and that bystander affiliation reduced redirection probability.

Even though both spontaneous and solicited third party affiliation occurred more frequently in absence of reconciliation in bonobos (Prediction 3a supported), the *Relationship Repair Hypothesis* had to be ruled out because the affiliating bystander and former aggressor did not share a strong bond (Prediction 3b not supported; Table 3). Among the aggressor's features considered, the only factor significantly influencing the frequency of spontaneous third party affiliation was the rank (Table 3), probably as part of a risk assessment strategy adopted by the bystander. Bystanders may be less inclined to get involved in post-conflict dynamics not to jeopardize their own relationship with a high-ranking group member. The use of third party affiliation as a relationship-repair mechanism has also been excluded in mandrills [42] and in the bonobo group studied by Clay and de Waal [22] because the triadic affiliation was not predicted by the kinship or relationship quality linking the bystander with the aggressor. However, as discussed later on, the higher frequency of third-party affiliation in absence of reconciliation may inform a different type of substitution not necessarily involving the bystander-aggressor relationship.

The *Consolation Hypothesis* is fully supported for spontaneous third party affiliation but not for solicited triadic contacts. Hence, from now on, we can refer to spontaneous third party affiliation as “consolation”, thus using its functional meaning. Consolation was affected by victim-bystander relationship quality (Table 4), being consolation preferentially offered to the victims sharing a close bond with the affiliating bystander (Prediction 4a supported; Figure 1). Additionally, post-conflict victim's scratching rates, informing anxiety, were reduced after consolation (Prediction 4b supported; Figure 2). This finding clarifies that third-party affiliation in bonobos works in reducing post-conflict anxiety in the victim, as also suggested by Clay and de Waal [22] in their study on two other groups of the same species.

This result also supports a previous work by Fraser et al. [17], who found that in chimpanzees spontaneous third party affiliation reduced victim's anxiety-related behaviours thus being consistent with the function of consolation in humans. For example, comfort provided to children by parents (e.g. via body contact) works in reducing infant cry, a clear distress signal [47]–[49].

Solicited triadic contacts did not have a consolatory function. They were not distributed according to the relationship quality of the individuals involved in the post-conflict affiliation (Prediction 4a not supported; Table 4). Moreover, solicited affiliation did not lead to a reduction of anxiety-related scratching in the victim (Prediction 4b not supported; Figure 2).

Both consolation and solicited affiliation played a significant role in victim protection, thus supporting the *Victim-Protection Hypothesis*. In fact, further attacks by group members were significantly reduced once the bystander had affiliated with the victim (Prediction 2 supported; Figure 3). This result cannot be easily compared with any other analogous outcome from previous studies on the bonobo, because in our knowledge no other previous studies have analyzed the frequency of further attacks by group members toward the former victim of an aggression.

One of the most conservative interpretations we can provide to this result is that the proximity serves as a deterrent for other group members to perform other attacks on the victims. Indeed, the fact that the protection works both in spontaneous (“motivated”) and solicited affiliation supports the idea that protection can be a byproduct of mere spatial proximity more than of social closeness, as it can also be hypothesized. Victim protection - found for both consolation and solicited affiliation - probably derives from two different driving forces: the self-oriented behaviour of the victim asking for help (solicited affiliation) and the prosocial behaviour of the third party providing comfort (consolation).

While the reduction of repeated attacks applied to solicited affiliation as much as to consolation, anxiety decrease only applied to the latter (Figure 2). Hence, in this case anxiety relief cannot be considered as the mere by-product of risk reduction - related to the lower probability of being re-attacked -because both solicited affiliation and consolation provided this benefit. Instead, anxiety alleviation can be related to consolation because only the spontaneous gesture appeared to be able to reduce anxiety-related behaviours to baseline levels. The spontaneous comforting gesture relying on “the consoler's motivational autonomy” (*sensu* de Waal [50]), may hold a greater significance to the victim as it is initiated by the consoler who, most probably, is an individual the victim shares a good relationship with.

In the study group, the higher frequency of consolation found in absence of reconciliation (the primary mechanism to reduce post-conflict anxiety [11]) indicates bystander's sensitivity to the victims' need for consolation when antagonism is left unresolved and the victim is still suffering anxiety. In this respect, consolation substitutes reconciliation in its function of reducing victim's anxiety but not in its function of restoring the relationship between the victim and their aggressor.

According to the present study, consolation comes to a greater extent from kin, then from “friends” (individuals strongly bonded to the consoler) and, lastly, from “acquaintances” (individuals weakly bonded to the consoler). Indeed, bonobo consolation appeared to follow the gradient (Figure 1) of empathy described in humans (lower between weakly bonded individuals, higher between tightly bonded ones, and highest in kin [4]). This and other pieces of information, described below, suggest that the comforting gesture may be driven by empathy-related mechanisms even if indirect benefits for the consoler (e.g., lower probability of further aggression in the group, maintenance of a good relationship with the victim) cannot be excluded.

Previous reports [17], [18] linked chimpanzee consolation (preferentially directed toward “friends” and kin) to sympathetic concern, an empathy related response [51], [52]. In humans, different clues link consolation and empathy. Similar to consolation, yawn contagion – proposed as an empathy related behavior (see Guggisberg et al. [53] for an extensive review) - is greatest in response to kin, then friends, then acquaintances [5]. Furthermore, individuals with autism spectrum disorders, involving empathy impairment [54], are unable to console and be infected by others' yawns [55], [56]. Additionally, in humans both comforting behavior and empathic abilities are age sensitive, increasing with age in both frequency and type of targets (cf. [8], [57]). For example, children first comfort family members and then other children, especially when hurt [8]. Recent data on young children of 1 year [58] and data on young bonobos [22] indicate that empathy tendencies can emerge younger than previously assumed. The fact that consolation and empathic abilities develop in tandem may inform their interrelation, suggesting that they may partly share the neuronal circuits underpinning emotional and social development. Indeed, there is increasing evidence that the areas underpinning empathy processing in humans have homologous areas in non-human primates [59]. Interestingly, it has been recently demonstrated that, as consolation, yawn contagion in bonobos is more likely to occur between friends and kin [60]. The link between consolation and empathy in apes could be further supported by experimental and naturalistic studies correlating consolation with phenomena of empathic sensorimotor resonance (e.g. yawn contagion and rapid facial mimicry [61]-[65]).

The consolation behavior in apes and humans are similar in the patterns used (e.g. touch/pat, embrace, and food/object sharing [1], [11], [21], [24], [66]), in the produced effect (anxiety reduction), and in distribution (relationship quality dependent). Hence, is it legitimate to hypothesize that in *Homo* and *Pan* – the last ape lines to have separated around 5–7 mya - consolation has arisen from different proximate factors, thus resulting from a phenomenon of convergent evolution? According to Darwin's principle of parsimony “*natural selection is continually trying to economize every part of the organization*” [67]. Applying this principle to consolation, de Waal [1] observed that “*if two related species show similar behavior under similar circumstances, the psychology behind their behavior is likely similar, too*”. In conclusion, if we accept that consolation is supported by empathic abilities in humans, we should be ready to accept the same for non human apes.

## Methods

### Ethics statement

This study was approved by University of Pisa (Animal Care and Use board). Since the study was purely observational the committee waived the need for a permit. The study was conducted with no manipulation of animals.

### Study site and group

We observed a group of bonobos over 10 years (2000–2009) in non-consecutive periods. Data were gathered at the Apenheul Primate Park, Apeldoorn, The Netherlands.

The animals were housed in an enclosure with both an indoor and outdoor facility (about 230 m^2^ and 5000 m^2^, respectively) and could move freely from the indoor to the outdoor enclosure after the first feeding session (at about 9:00 AM), and received abundant food (pellets, vegetables, fruits, rice and nuts, that were scattered on the ground) three times a day at 9:00 AM, 12:45 PM, and 5:00 PM. Water was available *ad libitum* and environmental enrichments were provided in the form of fresh branches, rice, and nuts scattered on the grass to encourage foraging activity, and renewal of the equipment in the indoor facility. Sometimes seeds and a wooden block with holes filled with honey, syrup were also furnished. No stereotypic or aberrant behaviors were observed during the entire period of data collection.

The demographic composition of the group varied across the study periods due to births, deaths and removals owing to management purposes. Over time the group consisted of 8–12 individuals. Kinship and age were known. Table 1 describes sex, age, and kinship of individuals, and group composition in every period of observation. The definition of age classes is consistent with [22].

### Data collection

Data collection involved four periods: A (July-October 2000), B (April-July 2002), C (September 2002-June 2003), and D (August-October 2009). We gathered 1,674 hours of live observations, performed daily over 6-hr periods, in both the morning and the afternoon. The coded data can be available for further validation. Data were collected by six students (two per period), trained by the same person (E.P.) until inter-observer reliability reached 95% in terms of identification of affiliative patterns, aggression patterns, affiliative contact initiators, and self-scratching.

Data were collected via 30-min focal observations (except for Post-Conflict and Matched Control observations, which lasted 10 min as described below). All agonistic interactions among individuals were collected by sampling all occurrences [68]. Agonistic interactions were identified as low when agonistic encounters included threats and chase-fleeing and as high when they also included physical contacts (such as biting, slapping, pushing, pulling, stamping, or brusque rushing) and fear reactions (screaming, bared teeth, or urination) [21].

For each agonistic interaction, opponents' identities were recorded and victims were followed as the focal individual for a 10-min Post-Conflict focal observation (PC). Matched Control focal observations (MC), in absence of agonistic interactions, were conducted the day after the conflict at the same time as the original PC [21]. We started observing the victim during the 10 min before the MC when the opponents had the opportunity to physically interact [45], [69]. Polyadic interactions (involving >2 opponents) were split into dyadic components [70]. For both PCs (10 min) and MCs (10 min) we recorded 1) starting/ending time (minute), 2) affinitive behaviors (grooming, touching, contact-sitting, food-sharing, social play, socio-sexual interactions, embracing, and kissing [27]) between the victim and other group members (Table 5), 3) victim's rough scratching bouts (self-directed behavior), 4) identity of individuals interacting with the victim, 5) time of each interaction (minute). Repeated attacks towards the victim performed by all group members (including the former aggressor) were recorded in the 10-min time window following the first aggression (if no affiliative contact occurred) and in the 10-min following the solicited or spontaneous contact.

**Table 5:** Ethogram of bonobo affiliative behaviours.

BEHAVIORAL PATTERN	DESCRIPTION
**Social play**	Play performed by two or more individuals engaging in one or more activities, such as tickling, gentle grabbing, pirouetting, pulling and/or pushing, slapping, sliding, and rough and tumble. The full ethogram of play is published in Tacconi and Palagi (2009).
**Embrace**	An individual clasps another individual with the one or both arms
**Grooming**	Fur cleaning performed by individual to another via hands or mouth
**Touch**	Gentle contact made by and individual to another using the hand's palm and avoiding genital parts (mainly hand in hand or hand on back). Touch can include patting (repeated touch from an individual to another performed with flat hand).
**Kiss**	An individual makes a lip, mouth-open contact with another
**Sit in Contact**	Two individuals sitting in contact to one another
**Socio-sexual contacts**	Two individuals making contact with at least one of them using the ano-genital area. They can take the form of genito-genital rubbing, mounting, copulation (with mountings and copulations distinguished on the basis of evident penal intromission), genital touch, rump-rump rubbing, inspecting, and masturbation
**Food Sharing**	An individual takes part or all of the food from the hands of the possessors, without eliciting any possessor's threatening response.

During each focal (PC), the first affinitive contact following an aggression could occur between the victim and the aggressor (reconciliation), be directed by a “third party” (an individual other then the aggressor) toward the victim (spontaneous third party affiliation), or by the victim toward a “third party” (solicited third party affiliation). Specifically, when the bystander approached the victim and initiated the first affiliative contact interaction (Table 5) toward the victim the third party affiliation was defined as “spontaneous”. Instead, when the victim approached a bystander and initiated the first affiliative interaction (Table 5), the third party affiliation was considered as “solicited”. Simple proximity (two individuals staying at arm-length distance but not in contact) following an approach was not sufficient to identify third party affiliation. Cases in which the initiator was unclear were excluded from the analysis.

Self-scratching was recorded during PCs, MCs, and all other focals to be used as a behavioural indicator of victim's anxiety [71]–[76]. We defined self-scratching as a repeated movement of the hand during which the fingertips are drawn across the individual's fur. A new scratching bout was assigned when the scratched body part changed, or when scratching was resumed after more than 5 s. To check for possible variation of self-scratching before and after third party affiliation we used the scratching frequencies of each individual calculated as the scratching bouts over the minutes of actual observation (that is from the beginning of the focal to the consolatory event and from the consolatory event to the end of the focal). In case of PCs with no third party affiliation and MCs the scratching bouts were normalised over 10-min (PC/MC focal duration).

We extracted background information on the relationship quality among group members by calculating the baseline frequencies of affiliative interactions (Table 5; Video S1 by Francesca Coppola) recorded during focal observations [68], other than PCs and MCs. Frequencies were obtained by normalizing the behavioral bouts over the observation time (30-min).

### Operational definitions and statistics

We considered the following individual characteristics: age (adult or immature), sex, and rank. Individuals' rank was assessed for each observation period by entering decided conflicts into a winner/loser socio-matrix. Such socio-matrices were reordered via Matman 1.0 and three rank levels were recognized: high (if an animal's rank fell into the upper quartile or top 25%), low (if animal's rank fell into the lower quartile, bottom 25%), and medium (if an animal's rank fell into the interquartile, 25% below and above the distribution median) (Table 2).

Relationship characteristics included kinship and affiliation levels. We considered as kin-related individuals belonging to grandmother/mother/offspring dyads and siblings (*r*≥0.25). Affiliation levels were determined using all affinitive behaviors (Table 5) within each dyad. Affiliation rates across dyads (corresponding to the absolute number of bouts of affinitive behaviors listed in Table 5 weighted on observation hours) were arranged according to a decreasing order. Kin dyads, which showed the highest affiliation levels, were considered as the strongest bond category. After excluding kin pairs, we categorized the relationship quality of non kin-dyads as strong if their affiliation levels fell into the upper quartile and as weak if otherwise.

We analyzed a total of 555 PC-MC, including 179 episodes of spontaneous third party affiliation and 121 cases of solicited third party affiliation. Analyses were performed via SPSS 19.0.

In absence of reconciliation, we evaluated the influence of both spontaneous and solicited third party affiliation on either anxiety-related rough scratching [73], [74] or multiple attacks targeting the same victim.

To analyze the rough scratching distribution, we defined four different conditions: PC-no cont (PC with no contact between the victim and other individuals in absence of reconciliation), PC-spont (PC exclusively including spontaneous third party affiliation in absence of reconciliation), PC-sol (PC exclusively including solicited third party affiliation in absence of reconciliation), MC (Matched Control). To analyze the distribution of multiple aggressive interactions we defined three different conditions: PC-no cont (PC with no contact between the victim and other individuals), PC-spont (PC exclusively including spontaneous third party affiliation), PC-sol (PC exclusively including solicited third party affiliation). Owing to the small sample size (N≤10) both analyses were run via the non-parametric Friedman's test. The Dunnett's test was used as Friedman's post-hoc for paired (k = 2) comparisons. Sample size and animals differed across tests because in each analysis we could include only individuals meeting all conditions [77].

The Wilcoxon matched-pair, signed-ranks test [77] was employed to assess differences between the number of attracted and dispersed pairs and to compare the frequency of either spontaneous or solicited third party affiliation in presence and absence of reconciliation, and in case of low and high intensity aggression. Non-parametric statistics was necessary due to the small sample size and/or deviation from normality (Kolmogorov-Smirnov: p<0.05). Exact values were selected following Mundry and Fischer [78].

Via LMM we evaluated the effect of individual characteristics of participants and relationship characteristics between opponents (either victim or aggressor) and third-parties (fixed factors) on the frequency of either spontaneous or solicited third party affiliation. We performed four tests: victim-third party relationship/features and frequency of either spontaneous or solicited third party affiliation; and aggressor-third party relationship/features and either spontaneous or solicited third party affiliation frequency.

Spontaneous and solicited third party affiliation frequencies were entered as dependent variables. They were calculated as the number of either spontaneous or solicited third party affiliation events normalized on the number of opportunities. Opportunities equaled the number of PCs in which one individual was the recipient, excluding those in which third-party was involved in the conflict (i.e. the aggressor or a supporter of either opponent) [24].

In all analyses we considered the following factors: intrinsic features of dyad's member (rank, sex, and age) and their paired combination per individual and per dyad (rank*sex, rank*age, age*sex), and relationship quality between dyad's members (affiliation bouts). Aggressor's, victim's, and third party's identities, and observation period were entered as random factors (nominal variables) (Table 2).

We tested models for each combination involving the variables of interest, spanning from a single-variable model to a model including all the fixed factors (full model). The variance of Covariance Parameter Estimates (CPE for each variable pair) is around zero (−0.01<CPE<0.10), indicating no correlation between the tested variables. To select the best model, we used the Akaike's Corrected Information Criterion (AICc), a measure for comparing mixed models based on the -2 (Restricted) log likelihood. The AICc corrects the Akaike's Information Criterion (AIC) for small sample sizes. As the sample size increases, the AICc converges to AIC. The AICcs of each set of tests were normally distributed (Kolmogorov-Smirnov test: ns). The model with a lower value of AIC was considered to be the best model. To avoid the increase of type II errors, factors were excluded from a model only if this improved the model fit by >2 AICc units [79]. The value of degrees of freedom is given by the effective sample size (N) minus the rank design matrix of fixed effects (X). The denominator degree of freedom is estimated by SPSS via Satterthwaite's approximation.

To compare dyadic third party affiliation frequencies as a function of the relationship quality, we used randomization tests (via Resampling Procedures 1.3 package by David C. Howell; 10000 permutations) accounting for data pseudo-replication [80]. The software provides a t value in the same way as in a standard t test, but calculates a p value as the proportion of randomized datasets that yield an even more extreme outcome.

## Supporting Information

Video S1 **Consolation via embrace in a bonobo group.** Use of the embrace as consolatory behaviour after a conflict in the bonobo colony (year 2009) of the Apenheul Primate Park (The Neatherlands). After a conflict between an adult female and a young females, an infant female console the victim by embracing her.
(AVI)

## References

[1] de Waal FBM (2012) Empathy in primates and other mammals. In: Decety J, ed. Empathy – From bench to bedside. Massachusetts, the MIT Press. pp 87–106.

[2] Singer T (2006) The neuronal basis and ontogeny of empathy and mind reading: review of literature and implications for future research. Neurosci Biobehav Rev 30: 855–863. 16904182 10.1016/j.neubiorev.2006.06.011

[3] Goleman DP (1995) Emotional intelligence: Why it can matter more than IQ for character, health and lifelong achievement. New York: Bantam Books.

[4] Preston SD, de Waal FBM (2002) Empathy: Its ultimate and proximate bases. Behav Brain Sci 25: 1–71. 12625087 10.1017/s0140525x02000018

[5] Norscia I, Palagi E (2011) Yawn contagion and empathy in *Homo sapiens* *PLoS ONE* **6**, e28472 (doi:10.1371/journal.pone.0028472). PMC3233580 22163307

[6] Fujisawa KK, Kutsukake N, Hasegawa T (2006) Peacemaking and consolation in Japanese preschoolers witnessing peer aggression. J Comp Psychol 120: 48–57. 16551164 10.1037/0735-7036.120.1.48

[7] Burleson BR (1983) Social cognition, empathic motivation, and adults' comforting strategies. Hum Com Res 10: 295–304.

[8] Eisenberg N (1992) The caring child. Cambridge, MA: Harvard University Press.

[9] de Waal FBM, van Roosmalen A (1979) Reconciliation and consolation among chimpanzees. Behav Ecol Sociobiol 5: 55–66.

[10] Watts DP, Colmenares F, Arnold K (2000) Redirection, consolation, and male policing. How targets of aggression interact with bystanders. In: Aureli F, de Waal FBM, eds, Natural Conflict Resolution. Berkeley and Los Angeles: University of California Press. pp 281–301.

[11] Fraser ON, Aureli F (2008) Reconciliation, consolation and postconflict behavioral specificity in chimpanzees. Am J Primatol 70: 1–10. 10.1002/ajp.20608 18767113

[12] Wittig RM, Boesch C (2003) The choice of post-conflict interactions in wild chimpanzees (*Pan troglodytes*) Behaviour. 140: 527–1559.

[13] Wittig RM, Boesch C (2010) Receiving post-conflict affiliation from the enemy's friend reconciles former opponents. PLoS ONE 5: e13995 10.1371/journal.pone.0013995 21085592 PMC2981571

[14] Kutsukake N, Castles DL (2004) Reconciliation and post-conflict third-party affiliation among wild chimpanzees in the Mahale Mountains, Tanzania. Primates 45: 157–165. 15114477 10.1007/s10329-004-0082-z

[15] Palagi E, Cordoni G, Borgognini Tarli S (2006) Possible roles of consolation in captive chimpanzees (*Pan troglodytes*). Am J Phys Anthropol 129: 105–111. 16229027 10.1002/ajpa.20242

[16] Koski SE, Sterck EHM (2007) Triadic postconflict affiliation in captive chimpanzees: does consolation console? Anim Behav 73: 133–142.

[17] Fraser ON, Stahl D, Aureli F (2008) Stress reduction through consolation in chimpanzees. Proc Natl Acad Sci USA 105: 8557–8562. 18559863 10.1073/pnas.0804141105 PMC2438392

[18] Romero T, de Waal FBM (2010) Chimpanzee (*Pan troglodytes*) consolation: third party identity as a window on possible function. J Comp Psychol 124: 278–86. 20695659 10.1037/a0019144

[19] Cordoni G, Palagi E, Tarli S (2006) Reconciliation and consolation in captive western gorillas. Int J Primatol 27: 1365–1382.

[20] Mallavarapu S, Stoinski TS, Bloomsmith MA, Maple TL (2006) Postconflict behavior in captive western lowland gorillas (*Gorilla gorilla gorilla*). Am J Primatol 68: 789–801. 16847973 10.1002/ajp.20279

[21] Palagi E, Paoli T, Borgognini Tarli SM (2004) Reconciliation and consolation in captive bonobos (*Pan paniscus*). Am J Primatol 62: 15–30. 14752810 10.1002/ajp.20000

[22] Clay Z, de Waal FBM (2013) Bonobos respond to distress in others: consolation across the age spectrum. PLoS ONE 8(1): e55206 10.1371/journal.pone.0055206 23383110 PMC3559394

[23] Fraser ON, Koski SE, Wittig RM, Aureli F (2009) Why are bystanders friendly to recipients of aggression? Commun Integr Biol 2(3): 285–291. 19641753 10.4161/cib.2.3.8718 PMC2717543

[24] Romero T, Castellanos MA, de Waal FBM (2010) Consolation as possible expression of sympathetic concern among chimpanzees. Proc Natl Acad Sci USA 107: , 12110–12115. 10.1073/pnas.1006991107 PMC2901437 20547864

[25] Wobber V, Wrangham R, Hare B (2010) Bonobos exhibit delayed development of social behavior and cognition relative to chimpanzees. Curr Biol 20: 226–230. 20116251 10.1016/j.cub.2009.11.070

[26] Tan J, Hare B (2013) Bonobos share with strangers. PLoS ONE 8(1): e51922 10.1371/journal.pone.0051922 23300956 PMC3534679

[27] Kano T (1992) The last ape. Palo Alto: Stanford University Press.

[28] Palagi E (2006) Social play in bonobos and chimpanzees: implications for natural social systems and inter-individual. Am J Phys Anthropol 129: 418–426. 16323189 10.1002/ajpa.20289

[29] Paoli T, Palagi E, Borgognini Tarli SM (2006) Reevaluation of dominance hierarchy in bonobos (*Pan paniscus*). Am J Phys Anthropol 130: 116–122. 16353224 10.1002/ajpa.20345

[30] Paoli T, Palagi E (2008) What does agonistic dominance imply in bonobos? In: Furuichi T, Thompson J, eds, Bonobos: Behaviour, Ecology, and Conservation. New York: Springer-Verlag. pp 35–54.

[31] Furuichi T (2011) Female contribution to the peaceful nature of bonobo society. Evol Anthropol 20: 131–142. 22038769 10.1002/evan.20308

[32] de Waal FBM, Aureli F (1996) Consolation, reconciliation and a possible cognitive difference between macaques and chimpanzees. In: Russon AE, Bard KA, Taylor Parker S, eds, Reaching into thought: the minds of great apes. Cambridge, UK: Cambridge University Press. pp 80–110.

[33] Hare B, Wobber V, Wrangham R (2012) The self-domestication hypothesis: evolution of bonobo psychology is due to selection against aggression. Anim Behav 83: 573–585.

[34] Palagi E, Cordoni G (2012) The right time to happen: play developmental divergence in the two *Pan* species. PLoS ONE 7(12): e52767 10.1371/journal.pone.0052767 23300765 PMC3530486

[35] de Waal FBM (1987) Tension regulation and nonreproductive functions of sex in captive bonobos (*Pan paniscus*). Natl Geogr Res 3: 318–335.

[36] Fruth B, Hohmann G (2002) How bonobos handle hunts and harvests: why share food? In: Boesch C, Hohmann G, Marchant LF, eds, Behavioural diversity in chimpanzees and bonobos. New York, USA: Cambridge University Press..pp 231–243.

[37] Palagi E, Paoli T, Borgognini Tarli SM (2006) Immediate and delayed benefi ts of play behaviour: new evidence from chimpanzees (*Pan troglodytes*). Int J Primatol 27: 1257–1270.

[38] Paoli T, Tacconi G, Borgognini Tarli S, Palagi E (2007) Influence of feeding and short-term crowding on the sexual repertoire of captive bonobos (*Pan paniscus*). Ann Zool Fenn 44: 84–88.

[39] Rilling JK, Scholz J, Preuss TM, Glasser M, Errangi B, et al. (2011) Differences between chimpanzees and bonobos in neural systems supporting social cognition. SCAN (DOI 10.1093/scan/nsr017) PMC3324566 21467047

[40] Koski SE, Sterck EH (2009) Post-conflict third-party affiliation in chimpanzees: what’s in it for the third party? Am J Primatol 71: 409–418. 19206165 10.1002/ajp.20668

[41] Call J, Aureli F, de Waal FBM (2002) Postconflict third party affiliation in stumptailed macaques. Anim Behav 63: 209–216.

[42] Schino G, Marini C (2012) Self-protective function of post-conflict bystander affiliation in mandrills. PLoS ONE 7(6): e38936 10.1371/journal.pone.0038936 22715420 PMC3371020

[43] Cheney DL, Seyfarth RM (1989) Reconciliation and redirected aggression in vervet monkeys *Cercopithecus aethiops* . Behaviour 110: 258–275.

[44] Wittig RM, Crockford C, Wikberg E, Seyfarth RM, Cheney DL (2007) Kin-mediated reconciliation substitutes for direct reconciliation in female baboons. Proc Biol Sci 274: 1109–1115. 17301022 10.1098/rspb.2006.0203 PMC2124468

[45] de Waal FBM, Yoshihara D (1983) Reconciliation and redirected affection in rhesus monkeys. Behaviour 85: 224–241.

[46] Veenema HC, Das M, Aureli F (1994) Methodological improvements for the study of reconciliation. Behav Proc 31: 29–38. 10.1016/0376-6357(94)90035-3 24897415

[47] Jahromi LB, Stifter CA (2007) Individual differences in the contribution of maternal soothing to infant distress reduction. Infancy 11: 255–269. 10.1111/j.1532-7078.2007.tb00226.x 33412735

[48] Erlandsson K, Dsilna A, Fagerberg I, Christensson K (2007) Skin-to-skin care with the father after cesarean birth and its effect on newborn crying and prefeeding behavior. Birth 34: 105–114. 17542814 10.1111/j.1523-536X.2007.00162.x

[49] Ganda AJ, Fara Ibrahim L, Natchimutu K, Ryan CA (2011) No more tears? Maternal involvement during the newborn screening examination. Clin Pediatr (Phila) 50: 753–756. 21540279 10.1177/0009922811405516

[50] de Waal FBM (2008) Putting the altruism back into altruism: the evolution of empathy. Annu Rev Psychol 59: 279–300. 17550343 10.1146/annurev.psych.59.103006.093625

[51] Eisenberg N, Eggum ND (2009) Empathic responding: sympathy and personal distress. In: Decety J, Ickes W, eds. The social neuroscience of empathy. Cambridge: MIT Press. pp. 71–83.

[52] Decety J (2010) The neurodevelopment of empathy in humans. Devel Neurosci 32: 257–267. 20805682 10.1159/000317771 PMC3021497

[53] Guggisberg AG, Mathis J, Schnider A, Hess CW (2010) Why do we yawn? Neurosci Biobehav Rev 34: 1267–1276. 20382180 10.1016/j.neubiorev.2010.03.008

[54] Lombardo MV, Barnes JL, Wheelwright SJ, Baron-Cohen S (2007) Self-referential cognition and empathy in autism. PLoS ONE 2(9): e883 10.1371/journal.pone.0000883 17849012 PMC1964804

[55] Frith U (1989) Autism: Explaining the enigma. Oxford, England: Blackwell.

[56] Senju A, Maeda M, Kikuchi Y, Hasegawa T, Tojo Y, et al (2007) Absence of contagious yawning in children with autism spectrum disorder. Biol Lett 3: 706–708. 17698452 10.1098/rsbl.2007.0337 PMC2391210

[57] Light SN, Zahn-Waxler C (2011) Nature and forms of empathy in the first years of life. In Decety J, ed, Empathy: from bench to bedside. Cambridge, MA: MIT Press. pp 109–130.

[58] Davidov M, Zahn-Waxler C, Roth-Hanania R, Knafo A (2013) Concern for others in the first year of life: theory, evidence, and avenues for research. Child Dev Perspect 7: 126–131.

[59] Iacoboni M (2012) The human mirror neuron system and its role in imitation and empathy. In: de Waal FBM, Ferrari PF, eds. The primate mind. Cambridge: Harvard University Press. pp 32–47.

[60] Demuru E, Palagi E (2012) In Bonobos Yawn Contagion Is Higher among Kin and Friends. PLoS ONE 7(11): e49613 10.1371/journal.pone.0049613 23166729 PMC3498209

[61] Platek SM, Critton SR, Myers TE, Gallup GG Jr (2003) Contagious yawning: the role of self-awareness and mental state attribution. Cognit Brain Res 17: 223–227. 10.1016/s0926-6410(03)00109-5 12880893

[62] Platek SM, Mohamed FB, Gallup GG Jr (2005) Contagious yawning and the brain. Cognit Brain Res 23: 448–452. 10.1016/j.cogbrainres.2004.11.011 15820652

[63] Fujisawa KK, Kutsukake N, Hasegawa T (2006) Peacemaking and consolation in Japanese preschoolers witnessing peer aggression. J Comp Psychol 120: 48–57. 16551164 10.1037/0735-7036.120.1.48

[64] Zahavi D, Overgaard S (2012) Empathy without isomorphism: a phenomenological account. In Decety J, ed, Empathy: from bench to bedside. Cambridge, MA: MIT Press. pp 3–20.

[65] Mancini G, Ferrari PF, Palagi E (2013) Rapid facial mimicry in geladas. Sci Rep 3: 1527 10.1038/srep01527 23538990 PMC3610402

[66] Zahn-Waxler C (1990) Radke-Yarrow (1990) The origins of empathic concern. Motiv Emotion 14: 107–131.

[67] Darwin C (1859) On the origin of species by means of natural selection. London: John Murray.

[68] Altmann J (1974) Observational study of behaviour sampling methods. Behaviour 49: 227–265. 4597405 10.1163/156853974x00534

[69] Kappeler PM, van Schaik CP (1992) Methodological and evolutionary aspects of reconciliation among primates. Ethology 92: 51–69.

[70] de Waal FBM, van Hooff JA (1981) Side-directed communication and agonistic interactions in chimpanzees. Behaviour 77: 164–198.

[71] Maestripieri D, Schino G, Aureli F, Troisi A (1992) A modest proposal: displacement activities as an indicator of emotions in primates. Anim Behav 44: 967–979.

[72] Aureli F, de Waal FBM (1997) Inhibition of social behavior in chimpanzees under high-density conditions. Am J Primatol 41: 213–228. 9057966 10.1002/(SICI)1098-2345(1997)41:3<213::AID-AJP4>3.0.CO;2-#

[73] Baker KC, Aureli F (1997) Behavioural indicators of anxiety: an empirical test in chimpanzees. Behaviour 134: 1031–1050.

[74] Troisi A (2002) Displacement activities as a behavioral measure of stress in nonhuman primates and human subjects. Stress 5: 47–54. 12171766 10.1080/102538902900012378

[75] Palagi E, Norscia I (2011) Scratching around stress: hierarchy and reconciliation make the difference in wild brown lemurs (*Eulemur fulvus*). Stress 14: 93–97. 20666657 10.3109/10253890.2010.505272

[76] Sclafani V, Norscia I, Antonacci D, Palagi E (2012) Scratching around mating: factors affecting anxiety in wild *Lemur catta* . Primates 53: 247–254. 22278710 10.1007/s10329-012-0294-6

[77] Siegel S, Castellan NJJ (1988) Nonparametric statistics for the behavioral sciences. New York: McGraw-Hill.

[78] Mundry R, Fischer J (1998) Use of statistical programs for nonparametric tests of small samples often leads to incorrect P values: examples from animal behaviour. Anim Behav 56: 56–259. 10.1006/anbe.1998.0756 9710485

[79] Jaeggi AV, Stevens JMG, van Schaik CP (2010) Tolerant food sharing and reciprocity is precluded by despotism among bonobos but not chimpanzees. Am J Phys Anthropol 143: 41–51. 20310060 10.1002/ajpa.21288

[80] Manly BFJ (1997) Randomization, bootstrap and Montecarlo methods in biology. London: Chapman and Hall.

